# Perioperative evaluation of CT-based body composition as predictors of postoperative outcome following pancreaticoduodenectomy

**DOI:** 10.3389/fnut.2025.1576144

**Published:** 2025-07-28

**Authors:** Lina Cai, Sizhen Wang, Yehua Xie, Hengyu Zheng, Daojun Zhu, Yi Xiao, Xinbo Wang, Xianghong Ye

**Affiliations:** Research Institute of General Surgery, Jinling Hospital, Affiliated Hospital of Medical School, Nanjing University, Nanjing, China

**Keywords:** body composition, postoperative pancreatic fistula, pancreaticoduodenectomy, sarcopenia, visceral obesity

## Abstract

**Background:**

Although malnutrition is a concern for incremental morbidity in pancreatic surgery, there has been a lack of consensus on nutritional assessment and body composition suitable for prediction of postoperative complications following pancreaticoduodenectomy (PD). Our study was performed to assess whether perioperative CT-based body composition were predictors of morbidity after PD.

**Methods:**

231 patients who underwent PD between 2020 and 2024 were enrolled to evaluate perioperative body composition. Uni and multivariate logistic regression models were applied to analyze the correlation between major complications, clinically relevant postoperative fistula (CR-POPF) and body composition abnormalities.

**Results:**

For 231 patients, the incidence of sarcopenia and visceral obesity was 151 (65.4) and 97 (42.0). The incidence of complications, major complications and CR-POPF was 68.0, 33.3 and 10.8%. SMI was the only risk factor for complications [odds ratio (OR), 0.92, 95% confidence interval (CI), 0.85–1.00, *p* = 0.04]. Neither sarcopenia, visceral obesity nor the other body composition had a significant impact on major complications or CR-POPF, while the patients exhibited wide variation in body composition after the surgical trauma. Soft pancreatic texture was the exclusive independent prognostic factor for CR-POPF (OR, 3.23, 95% CI, 1.17–8.89, *p* = 0.02).

**Conclusion:**

Patients with depleted skeletal muscle mass were more likely to develop postoperative complications, while there was no association between perioperative sarcopenia or visceral obesity and major complications or CR-POPF. The study highlights that the highly homogenized and fully managed surgical quality may offset the negative effects of nutritional high-risk factors.

## Introduction

Despite remarkable advancements in perioperative care, 6-month morbidity in pancreaticoduodenectomy (PD) did not significantly improve with morbidity rates between 38 to 73%, while other more specific morbidity indicators, for example clinically relevant postoperative pancreatic fistula (CR-POPF) rates, showed a large variation (0–35%) (1). Furthermore, major surgical complications after PD impact patient recovery and delay the timing of adjuvant treatments with potential implications on the long-term prognosis in periampullary cancer (2, 3). Therefore, early identifying and intervening of populations at risk of complications is of the utmost importance.

Although malnutrition is a concern for incremental morbidity, mortality, and costs in pancreatic surgery, there has been no fundamental consensus on diagnostic criteria and nutritional assessment suitable for prediction of postoperative complications in PD (4, 5). More recently, the evaluation of the nutritional status of patients undergoing pancreatic surgery has been suggested considering the measurement of sarcopenia and visceral obesity at CT. Depleted lean muscle mass, known as sarcopenia in pancreatic cancer surgery provides prognostic value but, more importantly, may provide a basis for therapeutic prehabilitation (6). Sarcopenia can also serve as a useful predictor of pancreatic fistula risk in patients undergoing PD (7, 8). A few studies have revealed that visceral obesity is a risk factor for CR-POPF (9, 10). These results highlight the potential of sarcopenia and visceral obesity improve the incidence of major complications or pancreatic fistula after PD. However, variation in the methods of assessing and reporting sarcopenia or visceral obesity in this patient group has shown inconsistent and inconclusive results (11, 12). CT is now routinely incorporated into the preoperative evaluation and postoperative management for the pancreatic cancer patients. Every patient undergoing pancreatic surgery routinely has an abdominal CT imaging evaluation for abdominal infection, pancreatic fistula, abdominal effusion, and other abdominal conditions 1 week postoperatively in our hospital as well as in most high volume pancreatic centers. Most previous studies investigated only the impacts of preoperative body composition components on surgical outcomes, it is expected that the perioperative assessment of CT-based body composition may improve risk stratification after PD.

Therefore, we designed a retrospective cohort study with the aim of assessing whether perioperative evaluation of CT-based body composition were independent predictors of postoperative complications, especially CR-POPF following PD for periampullary tumors.

## Methods

### Patients and baseline characteristics

This retrospective study was conducted at Jinling Hospital, Affiliated Hospital of Medical School, Nanjing University. Data from all 231 patients who underwent PD between March 2020 and May 2024 were prospectively collected from a database and retrospectively analyzed. The retrospective body composition evaluation was collected at the prospective CT within 30 days before scheduled surgery and on postoperative day (POD) 7. The study protocol was approved by the Ethics Committee of the Jinling Hospital (approval no. 2024NZKY-038-02). Information on the characteristics and clinical courses of the patients was obtained from their medical records at a single institution. Written informed consent was obtained from all patients.

Inclusion criteria: (1) Age ≥18 years; (2) Patients pathologically or clinically diagnosed with pancreatic tumors, periampullary tumors, chronic pancreatitis, or other conditions requiring pancreaticoduodenectomy (PD); (3) Availability of two abdominal CT scan datasets with image quality meeting the requirements for body composition analysis, specifically: slice thickness ≤5 mm, no obvious motion artifacts, and clear visualization of key tissues such as the psoas major muscle, liver, and adipose tissue. Exclusion criteria: (1) Presence of massive ascites, pleural effusion, or limb edema before surgery; (2) History of diseases affecting muscle metabolism, such as myasthenia gravis, uncontrolled hyperthyroidism, end-stage chronic renal failure, etc.

**Figure 1:**
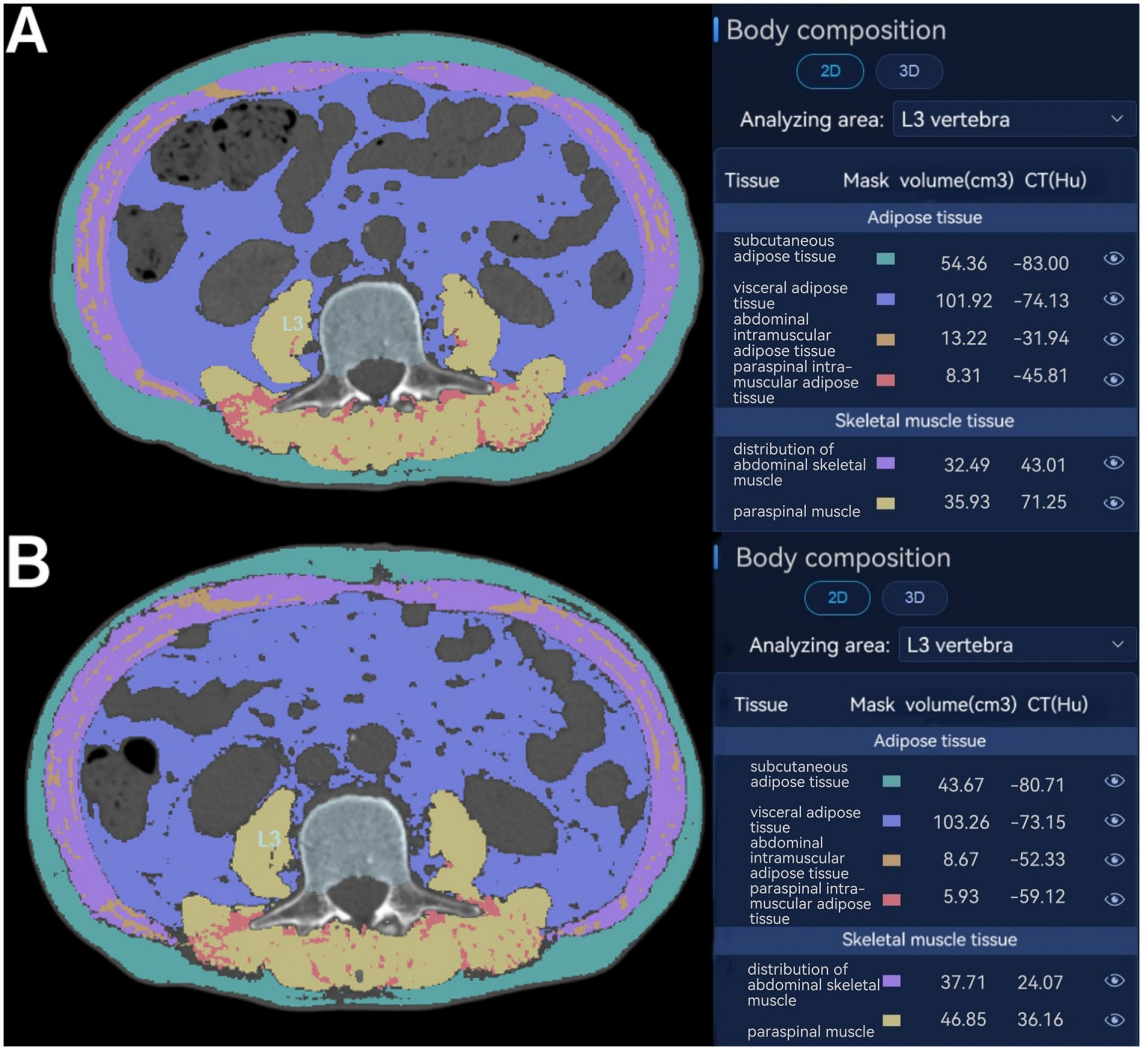
Assessment of body composition parameters on axial CT at the level of the inferior endplate of the third lumbar vertebra. Distribution of abdominal skeletal muscle (purple); abdominal intramuscular adipose tissue (brown); paraspinal muscle (yellow); paraspinal intramuscular adipose tissue (red); subcutaneous adipose tissue (green); visceral adipose tissue (blue) at CT before surgery **(A)** and postoperative day (POD) 7 **(B)**.

The following baseline variables were collected: age, sex, weight, body mass index (BMI), comorbidities (hypertension, coronary artery disease, diabetes, pulmonary and others), tumor characteristics, preoperative biliary drainage, operative time, blood loss, texture of the pancreas, and postoperative complications according to the Clavien-Dindo classification system. Preoperative blood tests contained white blood cell count (WBC), hemoglobin (Hb), C-active protein (CRP), albumin (Alb), aspartate aminotransferase (AST), alanine transaminase (ALT), total bilirubin (TBil), direct bilirubin (DBil), serum amylase (AMY), and CA19-9 (see Figure 1).

### Assessment of perioperative body composition at CT

All CT images were retrieved from the Picture Archiving and Communication System (PACS) at our institution. A clinically trained radiologists (Changsheng Zhou) analyzed the unenhanced CT scans with slice thickness of 1 mm, and another radiologist (Jing Zhong) double-checked the results. Both of them were blinded from the study design or patient information. The areas of subcutaneous adipose tissue (SAT), visceral adipose tissue (VAT), skeletal muscle at CT were evaluated using the Bone density CT imaging auxiliary detection software (HY-QCT, V2.5.0; Huiying Medical Technology Co., Ltd., Beijing, China). Two consecutive axial CT images at the level of the inferior endplate of L3 lumbar vertebra were processed and then averaged for each patient. The range of Hounsfield units (HU) was defined as −29 to +150 HU for skeletal muscle, −190 to −30 HU for SAT, and −150 to −50 HU for VAT (6). Using the HY-QCT software, the skeletal muscle area (SMA) (cm^2^), including the abdominal skeletal muscle and paraspinal muscle on the selected axial images was normalized to stature by dividing the muscle area by the patient’s height squared, and which is termed the skeletal muscle index [SMI = SMA (cm^2^)/height (m^2^)]. The visceral adipose tissue (VAT) (cm^2^) and the subcutaneous adipose tissue (SAT) (cm^2^) were also normalized for height in meters squared and are termed visceral fat index (VATI) and subcutaneous adipose tissue index (SATI). The absolute SMI/VATI/SATI variation, defined as the difference between values at POD7 and preoperative (∆SMI/∆VATI/ΔSATI), was also considered a potential prognostic marker for postoperative morbidity in Table 1.

**Table 1:** Descriptive statistics of the whole cohort and patients with or without complications.

Variables	Overall (*n* = 231)	Without complications (*n* = 74)	With complications (*n* = 157)	*p*-value*
Age, years	62 (54, 69)	62 (51, 67)	63 (54, 69)	0.34
Sex				0.47
Male, *n* (%)	142 (61.5)	43 (58.1)	99 (63.1)	/
Female, *n* (%)	89 (38.5)	31 (41.9)	58 (36.9)	/
Weight, kg	60.0 (55.0, 68.0)	60.0 (55.0, 65.8)	60.0 (54.5, 69.0)	0.83
BMI, kg/m^2^	22.23 (20.20, 24.39)	22.37 (20.52, 24.76)	22.15 (20.04, 24.22)	0.38
PNI	44.10 ± 7.12	43.32 ± 6.58	44.47 ± 7.35	0.25
Comorbidities				
Hypertension	76 (32.9)	21 (28.4)	55 (35.0)	0.32
Cardiac	10 (4.3)	3 (4.1)	7 (4.5)	1.00
Diabetes	44 (19.0)	18 (4.3)	26 (16.6)	0.16
Pulmonary	2 (0.9)	0 (0)	2 (1.3)	1.00
Other comorbidities	24 (10.4)	11 (14.9)	13 (8.3)	0.13
PBD, *n* (%)	42 (18.2)	10 (13.5)	32 (20.4)	0.21
NAT, *n* (%)	8 (3.5)	1 (1.4)	7 (4.5)	0.41
Disease Type				0.13
Malignant tumors, *n* (%)	190 (82.3)	59 (79.7)	131 (83.4)	/
Borderline tumors, *n* (%)	29 (12.6)	8 (10.8)	21 (13.4)	/
Benign diseases, *n* (%)	12 (5.2)	7 (9.5)	5 (3.2)	/
Operative time, min	240 (210, 310)	245 (210, 310)	240 (210, 320)	0.62
Estimated blood loss, ml	400 (300, 600)	400 (300, 525)	400 (300, 600)	0.53
Clavien–Dindo classifications, *n* (%)				
1	22 (9.5)	NA	22 (14.0)	/
2	58 (25.1)	NA	58 (36.9)	/
3A	46 (19.9)	NA	46 (29.3)	/
3B	29 (12.6)	NA	29 (18.5)	/
4	1 (0.4)	NA	1 (0.6)	/
5	1 (0.4)	NA	1 (0.6)	/
Major complications (CDC ≥ 3), *n* (%)	77 (33.3)	NA	77 (49.0)	/
CR-POPF, *n* (%)	25 (10.8)	NA	25 (15.9)	/
SMI, cm^2^/m^2^	40.40 ± 7.43	42.06 ± 6.38	39.62 ± 7.78	**0.019**
SATI, cm^2^/m^2^	38.47 (26.91, 53.96)	40.13 (27.36, 56.20)	38.46 (26.59, 52.25)	0.50
VATI, cm^2^/m^2^	37.90 (26.80, 56.12)	39.08 (28.72, 56.33)	36.99 (25.16, 56.07)	0.58
SMA, cm^2^	110.50 ± 24.72	114.32 ± 23.43	108.70 ± 25.18	0.11
SAT, cmP^2^	108.26 (74.13, 143.53)	110.65 (81.73, 143.70)	105.77 (73.55, 143.09)	0.50
VAT, cm^2^	102.63 (70.95,150.29)	107.28 (75.94, 152.74)	101.21 (68.05, 151.48)	0.67

*p values lower than 0.05 are indicated in bold. Values are median (interquartile range) or number (%). NA, Not applicable; BMI, Body mass index; PNI, Prognostic nutritional index; PBD, Preoperative biliary drainage; NAT, Neoadjuvant chemotherapy; CDC, Clavien-Dindo classifications; CR-POPF, Clinically relevant postoperative pancreatic fistula; SMI, Skeletal muscle index; SATI, Subcutaneous adipose tissue index; VATI, Visceral adipose tissue index; SMA, Skeletal muscle area; SAT, Subcutaneous adipose tissue; VAT, Visceral adipose tissue.

### Perioperative management and definitions

The indications for preoperative biliary drainage (PBD) in our institution include cholangitis, delayed surgery, and relief of jaundice in patients planned to receive neoadjuvant therapy (NAT). All the pancreatoduodenectomy procedures with or without pylorus preservation were performed or supervised by the same experienced surgeon (Xinbo Wang). A two-layer duct-to-mucosa pancreato-jejunostomy with Child technique was used for reconstruction. At the end of operation, two abdominal closed suction drains were then positioned to the bilio-jejunal anastomosis and the pancreato-jejunal anastomosis. Perioperative care was provided per the Enhanced Recovery After Surgery (ERAS) recommendations as we conferred before.^2^ Drainage tubes were removed when the routine evaluation CT scan at POD7 indicated no evidence of an encapsulated effusion in the abdominal cavity.

Postoperative complications were defined according to Clavien-Dindo classification (CDC), validated in pancreatic surgery (13, 14). Major complications were considered for CDC ≥ 3. Postoperative pancreatic fistula (POPF) was defined according to the International Study Group for Pancreatic Fistula (ISGPF) criteria as biochemical (grade A) or clinically relevant fistula (CR-POPF; grades B or C) (15). Postoperative mortality and morbidity were defined as occurring during hospital admission or within 30 d after discharge.

Sarcopenia was defined using the sex-specific SMI cutoff values of SMI < 43 cm^2^/m^2^ with BMI < 25 kg/m^2^ or SMI < 53 cm^2^/m^2^ with BMI ≥ 25 kg/m^2^ in males and SMI < 41 cm^2^/m^2^ in females (16). The thresholds for sarcopenia derived from Western population standards was really well studied in the Chinese population (17). Visceral obesity was defined as visceral adipose tissue (VAT) > 136 cm^2^ in men and > 95 cm^2^ in women as there were established for a cohort of patients similar to ours (18). As an indicator of nutritional status, prognostic nutritional index (PNI) was assessed using the following equation as described previously: PNI = 10 * serum albumin (g/dl) + 0.005 *total lymphocyte count in the peripheral blood (/mm^3^) (19).

### Statistical analysis

Statistical analysis was performed by SPSS 25.0 software (IBM Corp., Armonk, NY, United States). Continuous variables with normal distribution were presented as mean ± SD and compared with independent t test, Mann–Whitney U test was used when they showed skewed distribution and expressed as median (Interquartile range, IQR). For categorical variables were presented as absolute values and percentages and *χ*^2^ test or Fisher exact probability method was used for comparison between groups. Univariate and multivariate logistic regression model was applied to identify factors associated with major complications and CR-POPF in PD. Indices with statistically significant differences (*p* value< 0.05) in univariate analysis were included in the multivariate analysis, and gender, age, and BMI were also included as covariates in the multivariate analysis. The degree of association was estimated using corresponding odds ratios (OR) and their 95% confidence intervals. Statistical significance was considered at a *p* value < 0.05.

## Results

### Clinical characteristics of all patients

Between 2020 and 2024, there were 250 eligible patients underwent pancreaticoduodenectomy for periampullary tumors in this surgical registry in our hospital. Among these patients, 231 consecutive patients were selected based on the inclusion/exclusion criteria for this study. The reasons for exclusion were that a lack of preoperative CT performed within 30 days before surgery (*n* = 10) and postoperative CT at POD7 (*n* = 9). Among the 231 patients (142 males and 89 females) included, there were no missing data on the main variables in the analysis. Patient demographics are summarized in Table 1. The median age and BMI were 62 years and 22.23 kg/m^2^. Tumor patients accounted for 94.8%, the total morbidity rate was 68.0%, and the incidence of major complications (CDC ≥ 3) was 33.3%. Of these, 25 patients (10.8%) fulfilled the CR-POPF ISGPF definition postoperatively, while 206 patients (89.2%) had POPF 0/A. There was 1 case of postoperative mortality in this cohort in the figure.

Patients with complications had a lower SMI values (*p* = 0.019) than patients without complications. Other body composition-derived variables were not significantly different between the two groups. Moreover, after dichotomizing the cohort in complication and noncomplication patients, there were no significant difference on the baseline, surgical and histopathological data.

### Perioperative distribution of body composition

Patients exhibited wide variation in body composition after the surgical trauma (Table 2). In particular, at POD7, there was a drop in SAT and SATI, while SMI, SMA, VATI and VAT increased with respect to the preoperative values, although only VAT appeared somehow without reaching statistical significance. Moreover, the rate of sarcopenia has significantly decreased while the rate of visceral obesity has significantly increased postoperatively. These changes indicate the characteristics of postoperative short-term distribution of body composition after PD.

**Table 2:** Perioperative distribution of body composition of the whole cohort (*n* = 231).

Variables	Preoperative period	Postoperative period	*p*-value*
SMI, cm^2^/m^2^	40.40 ± 7.43	41.72 ± 6.38	**0.000**
SMA, cm^2^	110.50 ± 24.72	113.86 ± 21.30	**0.000**
SATI, cm^2^/m^2^	38.47 (26.91, 53.96)	37.43 (25.46, 51.06)	**0.000**
SAT, cm^2^	108.26 (74.13, 143.53)	101.34 (72.52, 134.22)	**0.000**
VATI, cm^2^/m^2^	37.90 (26.80, 56.12)	46.24 (32.57, 62.87)	**0.000**
VAT, cm^2^	102.63 (70.95,150.29)	110.81 (77.81, 145.52)	0.051
Sarcopenia, *n* (%)	151 (65.4)	136 (58.9)	**0.000**
Visceral obesity, *n* (%)	97 (42.0)	107 (46.3)	**0.000**

*p values lower than 0.05 are indicated in bold. Values are median (interquartile range). SMI, Skeletal muscle index; SMA, Skeletal muscle area; SATI, Subcutaneous adipose tissue index; SAT, Subcutaneous adipose tissue; VATI, Visceral adipose tissue index; VAT, Visceral adipose tissue.

### Risk factor analysis for complications, major complications and clinically relevant pancreatic fistulas

Risk factor analysis was conducted to determine the risk factors of complications, major complications and development of CR-POPF. Table 3 illustrates the risk factor analysis for complications. Sarcopenia and visceral obesity were noted in 111 (70.7%) and 66 (42.0%) of patients with complications and 40 (54.1%) and 31 (41.9%) of patients without complications, respectively. In univariate analysis, low SMI and sarcopenia were significantly related to the occurrence of complications. The low SMI was an independent risk factor for complications in multivariate analysis (odds ratio [OR], 0.92, 95% confidence interval [CI], 0.85–1.00, *p* = 0.04). However, visceral obesity and other body composition behavior were not statistically related to the occurrence of complications.

**Table 3:** Univariate and multivariate analyses of potential predictors associated with complications.

Variables	With complications (*n* = 157)	Without complications (*n* = 74)	Univariate(*p*-value*)	Multivariate		
				OR	95% CI	*p*-value*
Sex, male, *n* (%)	99 (63.1)	43 (58.1)	0.47	2.27	1.11–4.68	**0.03**
Age, years	63 (54, 69)	62 (51, 67)	0.30	1.00	0.98–1.03	0.83
BMI (kg/m2)	22.15 (20.04, 24.22)	22.37 (20.52, 24.76)	0.65	1.11	0.98–1.26	0.11
SMI (cm^2^/m^2^)	39.62 ± 7.78	42.06 ± 6.38	**0.02**	0.92	0.85–1.00	**0.04**
SATI (cm^2^/m^2^)	38.46 (26.59, 52.25)	40.13 (27.36, 56.20)	0.35			
VATI (cm^2^/m^2^)	36.99 (25.16, 56.07)	39.08 (28.72, 56.33)	0.63			
PBD, n (%)	32 (20.4)	10 (13.5)	0.21			
Albumin (g/L)	37.36 ± 6.46	35.88 ± 6.30	0.11			
Hemoglobin (g/L)	120.09 ± 18.54	123.81 ± 18.47	0.16			
Sarcopenia, *n* (%)	111 (70.7)	40 (54.1)	**0.01**	1.33	0.59–3.00	0.49
Visceral obesity, *n* (%)	66 (42)	31 (41.9)	0.98			

*p values lower than 0.05 are indicated in bold. Values are median (interquartile range) or number (%). Values are median (interquartile range) or number (%). OR, Odds ratio; CI, Confidence intervals; BMI, Body mass index; SMI, Skeletal muscle index; SATI, Subcutaneous adipose tissue index; VATI, Visceral adipose tissue index; PBD, Preoperative biliary drainage.

As exploratory analysis, the comparison of perioperative distribution of body composition between patients with and without major complications is shown in Table 4. There were not any independent risk factors for major complications (CDC ≥ 3) after PD in this cohort. Even those patients with sarcopenia or visceral obesity did not experience much more major complications. Regarding specific pancreatic complications, we also performed a risk factor analysis of CR-POPF development (Table 5). Univariable analysis showed statistical significance with high BMI, PNI, SMI and VATI, visceral obesity, lower ∆VATI between POD7 and before surgery, soft pancreatic texture, and high blood albumin in the occurrence of CR-POPF. After multivariate analysis, soft pancreatic texture was the exclusive independent prognostic factor for development of CR-POPF (OR, 3.23, 95% CI, 1.17–8.89, *p* = 0.02).

**Table 4:** Univariate analyses of potential predictors associated with major complications.

Variables	CDC < 3 (*n* = 154)	CDC ≥ 3 (*n* = 77)	*p*-value*	Odds ratio	95% CI
Sex, male, n (%)	92 (59.7)	50 (64.9)	0.45	1.25	0.71–2.20
Age, years	62.0 (52.8, 69.0)	64.0 (54.5, 69.0)	0.52	1.01	0.98–1.03
BMI (kg/m2)	22.37 ± 2.97	22.59 ± 3.04	0.60	1.03	0.94–1.12
PNI	44.16 ± 6.74	43.98 ± 7.87	0.86	1.00	0.96–1.04
SMI (cm^2^/m^2^)	40.35 ± 7.56	40.49 ± 7.22	0.89	1.00	0.97–1.04
SATI (cm^2^/m^2^)	38.43 (25.93, 54.94)	39.20 (28.67, 52.90)	0.89	1.00	0.99–1.01
VATI (cm^2^/m^2^)	37.74 (27.18, 55.36)	37.90 (26.75, 60.79)	0.32	1.01	0.99–1.02
∆SMI (cm^2^/m^2^)	0.02 (−0.03, 0.08)	0.03 (−0.04, 0.13)	0.39	2.77	0.28–27.85
∆SATI (cm^2^/m^2^)	−0.06 (−0.13, 0.03)	−0.05 (−0.15, 004)	0.83	1.20	0.23–6.29
∆VATI (cm^2^/m^2^)	0.18 (−0.04, 0.59)	0.12 (−0.04, 0.36)	0.18	0.62	0.31–1.24
Sarcopenia, *n* (%)	100 (64.9)	51 (66.2)	0.85	0.94	0.53–1.68
Visceral obesity, *n* (%)	61 (39.6)	36 (46.8)	0.30	1.34	0.77–2.33
Albumin (g/L)	36.76 ± 6.37	37.13 ± 6.60	0.69	1.01	0.97–1.05
Hemoglobin (g/L)	120.84 ± 19.13	122.17 ± 17.44	0.61	1.00	0.99–1.02

*p values lower than 0.05 are indicated in bold. Values are median (interquartile range) or number (%). CI, Confidence intervals; CDC, Clavien-Dindo classifications; BMI, Body mass index; PNI, Prognostic nutritional index; SMI, Skeletal muscle index; SATI, Subcutaneous adipose tissue index; VATI, Visceral adipose tissue index; ∆SMI/∆SATI/∆VATI defined as the difference between values (SMI/SATI/VATI) at POD 7 and preoperative.

**Table 5:** Univariate and multivariate analyses of potential predictors associated with clinically relevant postoperative pancreatic fistula (CR-POPF).

Variables	Non CR-POPF (*n* = 206)	CR-POPF (*n* = 25)	Univariate (*p*-value*)	Multivariate		
				OR	95% CI	*p*-value*
Sex, male, *n* (%)	126 (61.2)	16 (64)	0.78	0.71	0.22–2.29	0.57
Age, years	62 (53,69)	65 (54.5, 69.5)	0.60	0.30	0.98–1.08	0.30
BMI (kg/m^2^)	22.25 ± 2.88	24.02 ± 3.45	**0.007**	1.04	0.83–1.31	0.73
PNI	43.59 ± 6.60	48.28 ± 9.67	**0.003**	1.14	0.97–1.35	0.11
SMI (cm^2^/m^2^)	39.96 ± 7.36	44.02 ± 7.23	**0.01**	1.06	0.96–1.17	0.29
SATI (cm^2^/m^2^)	41.02 ± 20.50	49.45 ± 20.57	0.06			
VATI (cm^2^/m^2^)	36.67 (25.68, 53.32)	60.16 (32.83, 83.30)	**0.001**	1.01	0.98–1.04	0.64
∆SMI (cm^2^/m^2^)	0.03 (−0.03, 0.11)	−0.01 (−0.06, 0.05)	0.07			
∆SATI (cm^2^/m^2^)	−0.05 (−0.13, 0.04)	−0.04 (−0.12, 0.05)	0.88			
∆VATI (cm^2^/m^2^)	0.16 (−0.03, 0.54)	0.08 (−0.11, 0.29)	**0.04**	0.83	0.18–3.83	0.81
Sarcopenia, *n* (%)	136 (66.0)	15 (60)	0.55			
Visceral obesity, *n* (%)	80 (38.8)	17 (68)	**0.008**	0.80	0.19–3.37	0.76
Pancreatic texture, soft, *n* (%)	93 (45.1)	19 (76)	**0.006**	0.31	0.11–0.89	**0.03**
Albumin (g/L)	36.50 ± 6.14	40.05 ± 7.96	**0.01**	0.93	0.77–1.11	0.42
Hemoglobin (g/L)	120.80 ± 18.21	125.24 ± 21.25	0.26			

^*^p values lower than 0.05 are indicated in bold. Values are median (interquartile range) or number (%). OR, Odds ratio, CI, Confidence intervals; BMI, Body mass index; PNI, Prognostic nutritional index; SMI, Skeletal muscle index; SATI, Subcutaneous adipose tissue index; VATI, Visceral adipose tissue index; ∆SMI/∆SATI/∆VATI defined as the difference between values (SMI/SATI/VATI) at POD 7 and preoperative.

## Discussion

This retrospective study allowed us to evaluate perioperative distribution of body composition at CT in stratifying the risk of postoperative morbidity following pancreaticoduodenectomy (PD) for periampullary tumors. In particular, we have observed that skeletal muscle index (SMI) was an independent risk variable for postoperative complications. In univariable analysis, there were some associations between muscle and adipose behavior and clinically relevant postoperative pancreatic fistula (CR-POPF), while soft pancreatic texture was the sole independent risk factor for CR-POPF in the multivariable analysis. Surprisingly, no one of the perioperative body composition parameters were able to identify patients in a state of major complications after PD.

Sarcopenia and other body composition abnormalities are increasingly recognized not only as the progressive and generalized muscle and adipose disorders in pancreatic cancer, but also ones associated with a range of short-term and long-term oncological outcomes (20–23). This cohort study confirmed the important role of SMI as a prognostic factor for outcomes after PD. The prevalence of sarcopenia in pancreatic cancer patients range between 20 and 65% due to the heterogeneous groups of patients, difference in disease stage, and the different methods of measuring sarcopenia (11). Relationship between sarcopenia and outcome following PD is debated. The prognostic value of sarcopenia on postoperative complications and survival is clinically relevant as it can be objectively and reliably measured and is a potentially modifiable risk factor (24). It can be assessed by the routine preoperative staging computed tomography (CT) but its role in surgical outcome in particular the occurrence of POPF is still unclear and debatable. In some case, sarcopenia alone did not have any impact on the outcome of these complications post pancreatic surgery, while sarcopenic obesity was independently associated with 90-day mortality (25). The other two cohort studies also reported that sarcopenic obesity rather than sarcopenia itself, was associated with an increased risk of major complications after PD (26, 27).

The results of this study are surprising and controversial. Multiple studies have demonstrated that sarcopenia and sarcopenic visceral obesity exert negative prognostic effects on oncologic outcomes in PDCA. However, in this cohort, neither sarcopenia, visceral obesity, nor other body composition parameters showed a significant association with major complications or CR-POPF. In fact, the percentage of patients at high risk for malnutrition varies between the nutritional assessment metrics, and the patients assigned as high risk by these scores might be not significantly prone to more postoperative complications (5). There are some possible reasons why the present study found no association between perioperative body composition parameters and clinically relevant postoperative mortality and morbidity.

First, all patients in our study were managed according to ERAS principles. On admission, preoperative risk stratification for the occurrence of postoperative clinically relevant morbidity will be carefully evaluated, along with the identification of modifiable risk factors such as patient nutritional status. The ERAS programs integrated with multimodal prehabilitation strategies have been conceived to improve nutritional and functional capacity, reduce visceral obesity in cancer patients, minimize the surgical insult and reduce the extent of postoperative inflammation (28, 29). Our previous study confirmed that ERAS programs lower the risk of major complications in PD, and subsequently advance the time of initiation of adjuvant chemotherapy (2). Furthermore, high-risk patients identified through nutritional screening tools should be prioritized for perioperative nutritional support. Notably, subtle improvements in nutritional status and functional capacity may not be fully captured by CT-based body composition analysis performed before surgery, potentially limiting its sensitivity to short-term physiological changes (30). This discrepancy might lead to either underestimate or overestimate the value of malnutrition in risk stratification. Second, given the complexity of the procedure, the rate of postoperative morbidity following PD is usually higher when compared with other gastrointestinal operations. Any malnutrition or body composition abnormalities, however defined, has only a minor influence on the postoperative outcome. Hence, it is not surprising that the univariate analysis identified some body composition parameters including BMI, PNI, SMI, VATI and visceral obesity presence as independent determinants of CR-POPF onset while the effect of soft pancreatic texture remained the exclusive risk factor at the multivariate analyses (31, 32). Third, this study retrospectively recruited the consecutive patients nearly 4 years in a prospective surgical registry. The long span of retrospective recruitment in other studies might interfere with the analysis results, especially when the prognostic role of body composition was some extent not much clear in stratifying the risk of major complications and CR-POPF after PD. Furthermore, all the procedures in this study were performed or supervised by the same experienced surgeon. PD is highly invasive procedures with an inherent risk of complications. Taking this into consideration, the highly homogenized and fully managed surgical quality in the study greatly reduces the clinically relevant postoperative morbidity and mortality, which may also offset the negative effects of nutritional high-risk factors such as BMI, SMI, visceral obesity indicated in other studies. However, this would not contradict much of the evidence on the impact of malnutrition. We cannot emphasize the importance of nutritional management too much in the era of ERAS. There is also an urgent need for new and comprehensive nutritional assessment score and evaluation methods most suitable for prediction of postoperative complications in patients undergoing PD (12).

In our study, body composition parameters such as SMI and VATI increased after surgery, while SATI decreased, which may reside in the early postoperative proinflammation. Although the intraoperative management of intravenous fluid was standardized for each patient, thus reducing the risk of fluid overload in the extracellular compartment. Recently, studies have confirmed the usefulness of body composition measurements in assessing nutritional status and predicting prognosis in critically ill (33), post-gastrectomy (34), and pancreatic trauma patients (35). It was found that on day 3, 6, and 9 after PD, the extracellular water level based on bioimpedance vectorial analysis (BIVA) was significantly higher than the preoperative level (36). The increasing popularity of neoadjuvant therapy today may also interfere with the accuracy of skeletal muscle measurements (37). Early postoperative CT-measured sarcopenia and visceral obesity demonstrates an undiscernible correlation with an elevated risk of postoperative morbidity and mortality in the context of PD. The ready availability of sequential CT offers a valuable opportunity for body composition assessment and a potential surrogate for blood inflammatory biomarkers as well as sequential BIVA. However, the quality of assessment and interpretation must improve before the impact of body composition on treatment-related outcomes and survival can be assessed (38). It is well known this attitude to reduced tolerance to stressful events underlies how the sarcopenia and visceral obesity has been widely associated with the concept of frailty (39).

The current study has important limitations. First, CT has an innate uncertainty to whether the same cross-sectional area, such as L3 level used in the study, captures treatment effects, especially if strength exercise intervention cooperated with nutritional support in prehabilitation program mainly involves large muscle groups in the upper and lower extremities when measuring body composition (40). Considering fat mass, previous study has reported that a single CT image slice may not accurately predict adipose tissue changes during weight loss in cancer patients (41). Second, the application of our perioperative absolute variation of body composition at CT should be further investigated and validated with different body composition analyzers and proinflammatory markers. Dedicated software to process the images and interpretation from a trained radiologist are required. Certain body conditions provoking extreme hyperhydration or dehydration in the early postoperative proinflammatory status after PD may bias the assessment of muscle mass. Third, we only evaluate the risk stratification of muscle mass quantification at CT without assessing muscle strength, which is increasingly being recognized as a risk factor for major complications after gastrointestinal surgery (42) or PD (7). Future prospective studies may more accurately assess sarcopenia by utilizing both imaging and clinical data, such as frailty. Finally, it was restricted to one center that specialized in PD based on a homogenous Chinese population, and the results may have limited applicability to other institution. In future multicenter prospective studies, we will include more patients to mitigate this limitation and further assess the utility of the respective thresholds using both Eastern and Western criteria.

## Conclusion

To our knowledge, this is the first study to retrospectively evaluate perioperative body composition behavior as an important prognostic marker and its relationship with nutritional status, in patients who underwent pancreaticoduodenectomy surgery. We did not see an association between sarcopenia or visceral obesity based on CT and major complications or CR-POPF, but there was a relation with the skeletal muscle index and postoperative complications. The study provides the most robust evidence to date that the highly homogenized and fully managed surgical quality in the era of ERAS greatly reduces the clinically relevant postoperative morbidity and mortality following PD, which may also offset the negative effects of nutritional high-risk factors such as BMI, SMI, visceral obesity indicated in previous studies. Future prospective studies may not only more accurately assess sarcopenia and visceral obesity, but also provide a basis for therapeutic prehabilitation by utilizing both imaging and functional assessment data for prediction of postoperative complications in patients undergoing PD.

## Data Availability

The raw data supporting the conclusions of this article will be made available by the authors, without undue reservation.
